# Effects of sildenafil treatment on thermogenesis and glucose homeostasis in diet-induced obese mice

**DOI:** 10.1038/s41387-018-0026-0

**Published:** 2018-03-13

**Authors:** Kornelia Johann, Marlen Colleen Reis, Lisbeth Harder, Beate Herrmann, Sogol Gachkar, Jens Mittag, Rebecca Oelkrug

**Affiliations:** Department of Internal Medicine I, Group of Molecular Endocrinology, University of Lübeck, Ratzeburger Allee 160, 23562 Lübeck, Germany

## Abstract

Stimulation of thermogenic pathways appears to be a promising approach to find new ways of tackling metabolic diseases like obesity and diabetes mellitus type 2. Thermogenic, weight reducing and insulin sensitizing effects of phosphodiesterase 5 (PDE 5) inhibitors have recently been postulated, suggesting that modulators of endogenous cGMP signaling have the therapeutic potential to treat metabolic disorders. However, most studies have been performed *in vitro* or in animals that were not glucose intolerant. We, thus, aimed to test the metabolic effects of the PDE 5 inhibitor sildenafil by treating diet-induced obese (DIO) mice orally for 8 days. Surprisingly, our results revealed no changes in body temperature, brown adipose tissue (BAT) thermogenesis and gene expression in BAT and inguinal white adipose tissue (iWAT), thus excluding a thermogenic or 'browning' effect of sildenafil in preexisting obesity. In contrast, sildenafil-treated DIO mice displayed changes in liver metabolism and glucose homeostasis resulting in impaired glucose tolerance (*P* < 0.05), demonstrating for the first time an unfavorable metabolic effect of increased hepatic cGMP signaling in obesity. As sildenafil is commonly prescribed to treat pulmonary arterial hypertension and erectile dysfunction in diabetic and/or obese patients, follow up studies are urgently required to re-evaluate the drug safety.

## Introduction

Recently, brown adipose tissue (BAT) has frequently been postulated as a novel target in the fight against obesity and diabetes in humans^1^, as it is capable of burning high amounts of triglycerides and releasing the resulting energy in form of heat^2^. Since adult humans possess only minor amounts of brown adipocytes in the supraclavicular region, the discovery of signaling pathways that lead to the induction of brown-like (beige/brite) adipocytes within white adipose tissue (WAT)—so called 'browning'—appeared to be a hallmark in anti-obesity therapy^3^. However, convincing evidence is still lacking that induction of brown-like adipocytes significantly improves whole-body energy expenditure in mice or humans^4^.

Activation of the NO/cGMP axis is essential for brown adipocyte development and function^5^, and has recently been shown to induce browning of inguinal WAT (iWAT) in mice^6^. Accordingly, a pharmacotherapy that elevates cGMP levels in obesity might be a promising therapeutic approach. Sildenafil is a phosphodiesterase 5 (PDE 5) inhibitor that increases cGMP levels and is widely used to treat pulmonary arterial hypertension (PAH) and erectile dysfunction (ED), especially in diabetic neuropathy occurring frequently in obesity^7,8^. Consequently, initial studies *in vitro*, in lean animals as well as pre-diabetic humans, suggested that sildenafil might have beneficial metabolic effects via 'browning' of iWAT^6,9^. However, little is known on the effects of sildenafil in a glucose-intolerant condition. Our study thus aims to investigate the consequences of sildenafil treatment on thermogenesis and glucose homeostasis in diet-induced obese (DIO) mice with a focus on a possible involvement of brown/brown-like adipocytes.

## Materials and Methods

### Animal husbandry

Experiments were performed on male wild-type C57BL/6NCr (Charles River, Germany) mice at the age of 3–5 months (*n* = 8 independent animals per group). The mice were housed in groups of 4 animals at an ambient temperature of 22 ± 1 °C, on a constant 12-hour light/dark cycle and were fed ad libitum. All animal procedures were approved by the MELUR Schleswig-Holstein, Germany.

### Experimental setup

Beginning at the age of 12 weeks, the animals were fed a high-fat diet (HFD, 60% fat, DC 12492, Research Diets, USA). After 6 weeks on HFD, the first metabolic analysis was performed, which included a glucose tolerance test (GTT, 1.5 g/kg body weight i.p.), infrared thermography (T335, FLIR, Sweden), and rectal thermometry (BAT-12, Physitemp, USA). After 1 week of recovery, the animals were randomly assigned to control (*n* = 8) or sildenafil (*n* = 8) group. The sildenafil group was orally treated with 400 µg/mL sildenafil citrate (Stada®) dissolved in acidified tap water (pH 3.0), whereas the control group received only acidified tap water^10^. Body weight, food, and water intake were measured daily. Daily water intake per mouse was 2.87 mL ± 0.13 mL, which equals to an uptake of 1.15 mg of sildenafil per day and mouse. On the basis of the average body weight of sildenafil-treated mice of 43.3 ± 2.5 g the daily sildenafil dosage was extrapolated to 26.6 mg/kg. Infrared pictures were taken on treatment day 5 and the GTT was repeated on treatment day 6. After 2 days of recovery, the animals were sacrificed and blood and organ samples were collected.

### Quantitative real-time PCR (qPCR)

RNA isolation and quantitative real-time PCR of liver, iBAT, iWAT, and epididymal WAT (eWAT) were performed as described previously^11^.

### ELISA/EIA

Serum C-peptide 2 (EZRMCP2-21K, Millipore, Germany) and hepatic cGMP content (RPN226, GE Healthcare, UK) were measured using commercial kits according to the manufacturer’s instructions.

### Glycogen content and enzyme activity

Glycogen content, PEPCK, and PK enzyme activity of snap-frozen liver tissue samples were determined as described previously^12^.

### Histology

Haematoxylin and eosin staining was performed on 5 µM paraffin-embedded liver sections according to the manufacturer’s instructions (H&E fast staining solution, Carl Roth, Germany).

### Statistical analysis

GraphPad Prism 6 software was used to analyze the data. Variances between the groups were similar and appropriate tests were performed to analyze statistical differences between control and sildenafil-treated groups, as indicated in text and figure legends. Values are represented as mean ± s.e.m..

## Results

### Metabolic and thermogenic effects of sildenafil treatment

**Fig. 1:**
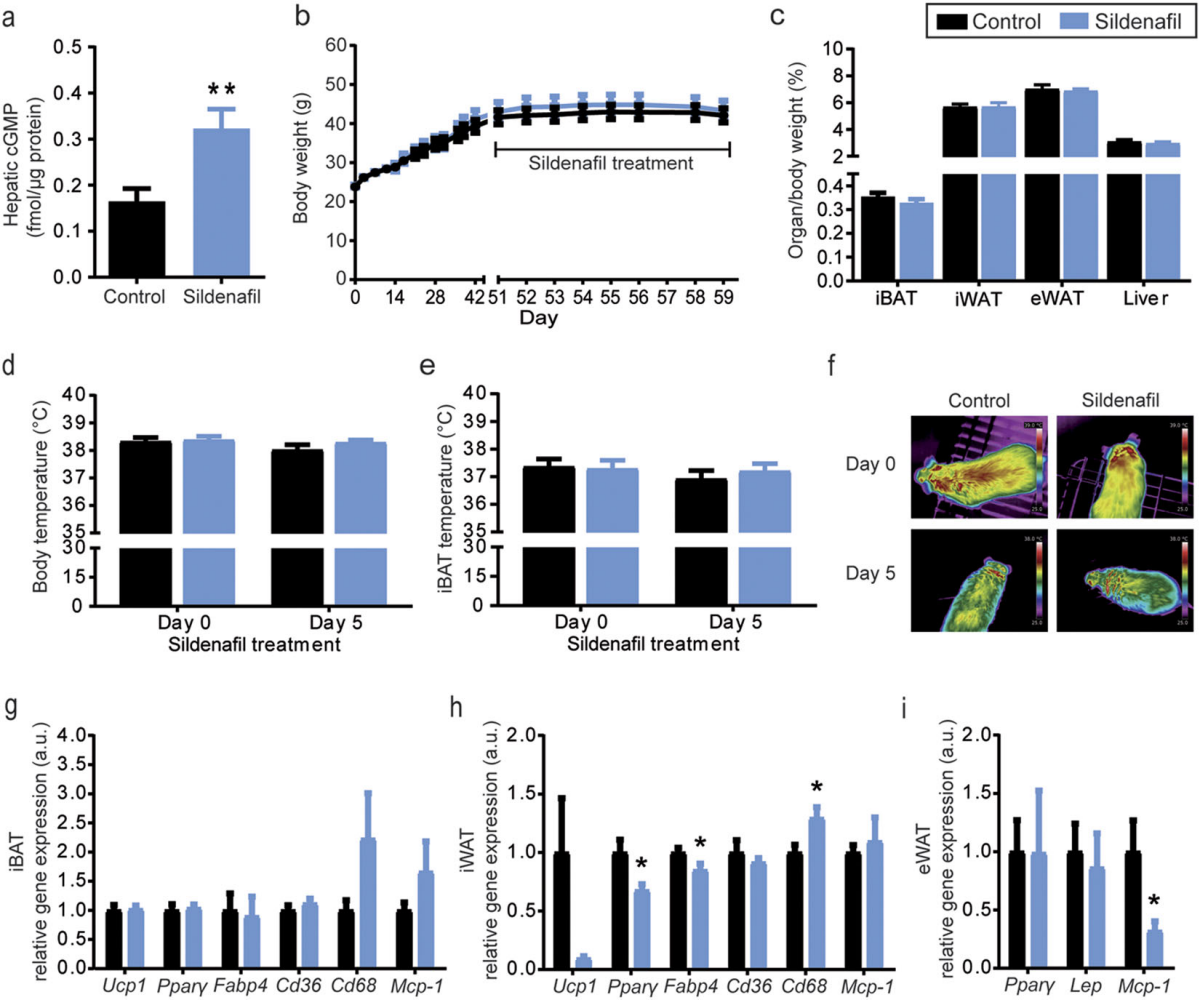
Sildenafil treatment does not lead to changes in body weight or thermogenesis of DIO mice. **a** Eight days of sildenafil treatment induced a significant elevation of hepatic cGMP levels in DIO mice (*P *= 0.0089). **b** However, body weight of control mice and mice treated with sildenafil was not affected by the treatment and **c** organ weights of iBAT, iWAT, eWAT, and liver (normalized to body weight) were similar. **d** Rectal body temperature was measured using a small-diameter thermocouple probe (accuracy: 0.1 °C) before (day 0) and during the sildenafil treatment (day 5). **e**–**f** In parallel, infrared thermography (sensitivity: 0.05 °C) of the iBAT area was performed on conscious animals and both were unchanged after 5 days of treatment. **g**–**i** In line, gene expression analysis in iBAT revealed no differences in expression of *Ucp1*, *Pparγ*, *Fabp4*, *Cd36, Cd68*, and *Mcp-1*, whereas in iWAT *Pparγ* (*P* = 0.0233) and *Fabp4* (*P* = 0.0425) were significantly downregulated and *Cd68* (*P* = 0.0201) upregulated. Furthermore, *Mcp-1* expression was significantly lower in eWAT after sildenafil treatment (*P* = 0.0406). *P < 0.05, **P < 0.01, unpaired, two-tailed t-tests with Welch-correction (**a, c, g**–**i**). Repeated measures two-way ANOVA following Bonferroni’s multiple comparisons test (**b, d**–**f**). Data are presented as mean ± s.e.m., *n* = 8. *Cd36* cluster of differentiation 36, *Cd68* cluster of differentiation 68, cGMP cyclic guanosine monophosphate, eWAT epididymal white adipose tissue. *Fabp4* fatty acid-binding protein 4, iBAT interscapular brown adipose tissue, iWAT inguinal white adipose tissue, *Lep* Leptin, *Mcp-1* monocyte chemoattractant protein-1, *Pparγ* peroxisome proliferator-activated receptor gamma, *Ucp1* uncoupling protein 1

Inhibition of PDE 5 by oral sildenafil treatment led to a 1.96—fold elevation of hepatic cGMP levels in DIO mice (Fig. 1a), confirming the efficacy of the treatment. However, it did not affect weight gain in preexisting obesity, resulting in similar body weights of sildenafil-treated and control animals at the day of sacrifice (treatment day 8, Fig. 1b). In addition, liver wet weight, as well as organ weights of selected fat pads, were unchanged (Fig. 1c) and food and water intake were similar (data not shown).

Although increased cGMP signaling is expected to lead to BAT activation and changes in body temperature regulation, we did not detect an effect of sildenafil treatment on body temperature (Fig. 1d) or iBAT temperature in DIO mice (Fig. 1e–f). Furthermore, gene expression analysis of important thermogenic and lipolytic marker genes in iBAT revealed no alterations in the thermogenic profile of brown adipocytes (Fig. 1g). Even more importantly, sildenafil did not induce browning of WAT as shown by gene expression analysis of browning markers in iWAT (Fig. 1h). The expression of monocyte chemoattractant protein-1 (*Mcp-1*), a chemokine that serves as marker for obesity-associated inflammation, was unchanged in iBAT, slightly elevated in iWAT, and significantly downregulated in eWAT of sildenafil-treated animals (*P* = 0.0406, Fig. 1g-i).

### Effects of sildenafil treatment on liver metabolism and glucose homeostasis

**Fig. 2:**
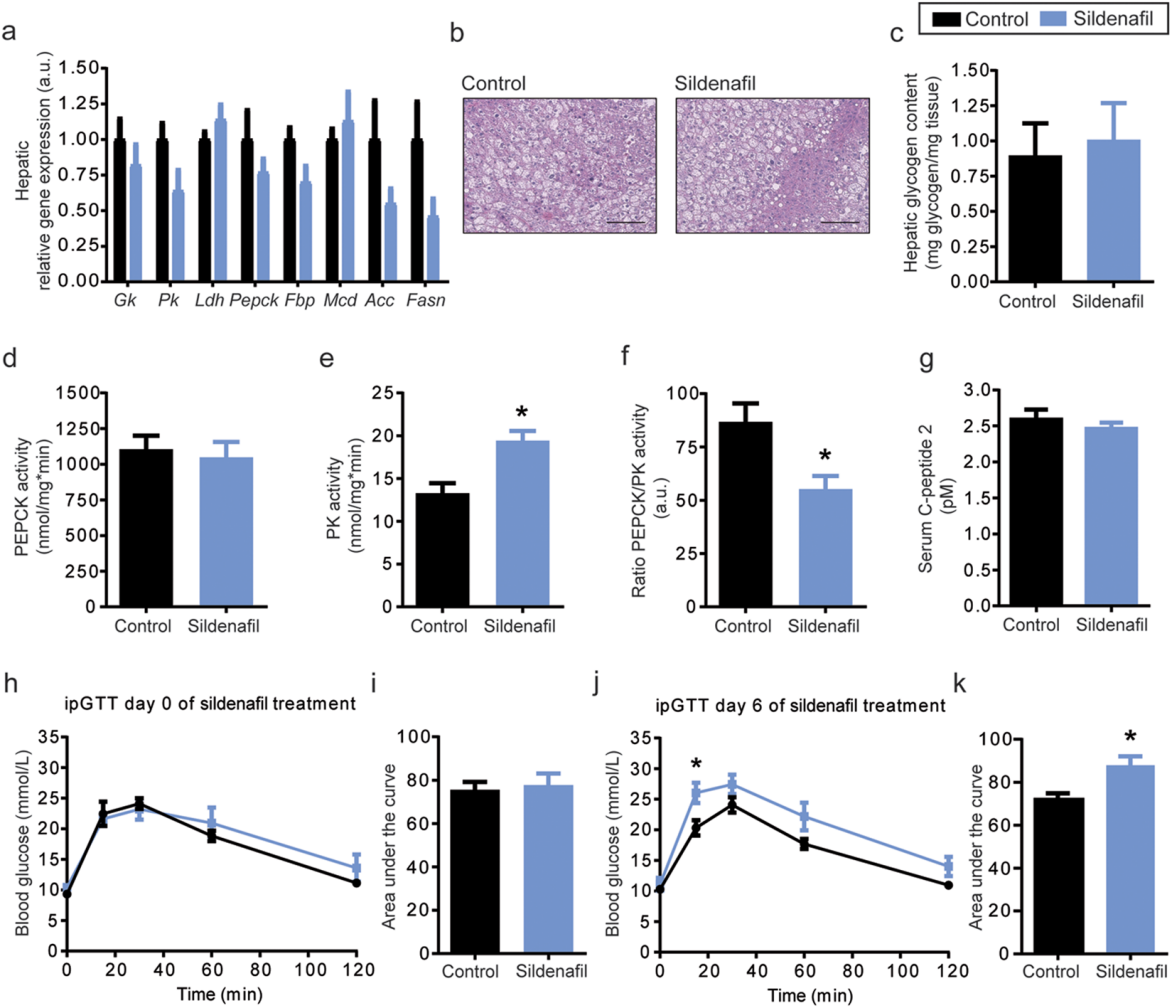
Sildenafil treatment impairs glucose tolerance in DIO mice. **a** Sildenafil treatment led to no changes in gene expression of important enzymes for glucose (*Gk*, *Pk*, *Ldh*, *Pepck, Fbp*) and fatty acid metabolism (*Mcd*, *Acc*, *Fasn*). **b** Furthermore, liver histology and **c** hepatic glycogen content were not affected by the treatment. **d**–**f** Although, sildenafil altered activity of an important enzyme for glycolysis (PK, *P* = 0.0021), but not for gluconeogenesis (PEPCK), still leading to a reduced PEPCK/PK ratio (*P* = 0.0106). **g** While serum C-peptide 2 levels were not affected by sildenafil treatment, **h**–**k** glucose tolerance was impaired on treatment day 6. Animals were fasted for 6 h before they received an intraperitoneal injection of glucose (1.5 g/kg body weight, day 0 and day 6). Blood glucose concentrations were measured in blood drawn from the tail vein using a commercially available glucometer (AccuCheck, Roche, Germany). *P < 0.05, **P < 0.01, unpaired, two-tailed *t*-tests with Welch-correction (**a**, **c**–**g**, **i**, **k**). *P < 0.05, repeated measures two-way ANOVA following Bonferroni’s multiple comparisons test (**h**, **j**). Data are presented as mean ± s.e.m., *n* = 8. *Acc* acetyl-coA carboxylase, *Fasn* fatty acid synthase, *Fbp* fructose-1.6-bisphosphatase, *Gk* glucokinase, ipGTT intraperitoneal glucose tolerance test, *Ldh* lactate dehydrogenase, *Mcd* malonyl-CoA decarboxylase, *Pepck* phosphoenolpyruvate-carboxykinase, *Pk* pyruvate kinase

We tested the consequences of increased cGMP signaling on liver metabolism (Fig. 2a), revealing that sildenafil treatment led to no alterations in gene expression of rate-limiting enzymes of glycolysis, gluconeogenesis, or fatty acid metabolism. However, enzyme activity assays demonstrated that sildenafil treatment induced a switch from hepatic gluconeogenesis to glycolysis, as indicated by a significantly higher activity of hepatic pyruvate kinase (PK, *P* = 0.0021, Fig. 2d–e) and a significantly lower PEPCK/PK ratio in sildenafil-treated DIO mice (*P* = 0.0106, Fig. 2f). However, the increase in PK activity did not affect hepatic glycogen content, and liver morphology was unaltered (Fig. 2b–c).

Although previous studies reported beneficial effects of increased cGMP levels on insulin signaling in vivo^9,13^, in our DIO mice we observed a reduction in glucose tolerance in mice after short-term treatment with sildenafil (Fig. 2h–k). While glucose tolerance of control mice did not change between day 0 and day 6 (mean AUC of control group, day 0 = 75.6 ± 3.6, day 6 = 72.7 ± 3.1, paired *t*-test, *P* = 0.5157), glucose tolerance in sildenafil-treated mice was significantly impaired after 6 days (mean AUC of sildenafil group, day 0 = 77.7 ± 5.4, day 6 = 87.7 ± 5.9, paired *t*-test, *P* = 0.0344). Unaltered serum levels of C-peptide 2 pointed to impairments in insulin sensitivity, like for example alterations in skeletal muscle glucose uptake rather than changes in insulin secretion (Fig. 2g).

## Discussion

This is the first study showing unfavorable metabolic effects of sildenafil treatment in an animal model of preexisting obesity and insulin resistance, although NO/cGMP signaling is known to improve substrate delivery to skeletal muscle by enhancing capillarisation^14^, stimulate muscular glucose uptake^15^ and attenuate endothelial dysfunction associated with insulin resistance^16^. The mechanisms how PDE 5 inhibitors influence glucose homeostasis are not completely understood, but recent studies suggested insulin sensitizing effects^13,16,17^. However, almost all studies on mice were either conducted on lean animals or before the onset of high-fat diet feeding^6,13^, which are conditions not reflecting obesity and insulin resistance. Due to the plethora of pathologies accompanying obesity, it is not surprising that different effects were observed in this study compared to previous findings in a more healthy condition. In humans, sildenafil (25 mg, 3x/day) enhanced insulin sensitivity after 3 months of treatment^9^; however, t1his study was also conducted on pre-diabetic patients. Other studies employing the PDE 5 inhibitor tadalafil showed somewhat improved β-cell function in metabolic syndrome of severe obese patients^17,18^, but overall no improvement in insulin resistance was found^18^. Taken together, our findings underline the importance of using appropriate metabolic model systems, as the resulting effects can differ substantially between healthy and pathological states.

Another aim of this study was to further unravel the role of cGMP signaling on brown/brown-like adipocytes in anti-obesity therapy. In line with previous studies we detected no effect on BAT mediated thermogenesis and *Ucp1* expression^6,13^. However, in contrast to previous findings in lean mice^6^, sildenafil did not induce browning of iWAT in DIO mice. These differences could be explained by the route of administration (drinking water vs. daily single injection), as the short half-life of sildenafil in rodents (0.4–1.3 h)^19^ leads to a high pulsatile effect in the injection model^6^. However, it is more likely that the pathologically obese state of DIO mice prevented iWAT browning, as it is also well established in mice and humans that browning is much harder to achieve under obese conditions^4,20^. Consequently, our study suggests that sildenafil might not be suitable to induce browning of WAT under obese conditions, although our dose of about 26.6 mg/kg/day is at the upper pharmacological range, even when taking into account the lower oral bioavailability in mouse (17%) compared to man (38%)^16,19^. Our results indicate, that DIO might lead to some form of drug resistance which was accompanied by a reduction in glucose tolerance. Furthermore, the tested dose of sildenafil might be responsible for the unfavorable metabolic effects observed in this study, and further dose-effect studies are urgently required—as sildenafil is not only used in short-term application to treat ED (up to 100 mg per occasion), but also prescribed long-term to patients with PAH (25 mg, 3x/day). Case studies even report patients with severe PAH or ED, which use higher doses of up to 240 mg/day or 1300 mg on single occasions^21^—doses high enough to reach a severely pharmacological state as in our study. Given that PAH and ED patients are often overweight, possible impairments of metabolic function in obesity by sildenafil need to be closely monitored in these patients.
